# A model approach to show that monocytes can enter microporous β-TCP ceramics

**DOI:** 10.1186/s12896-024-00857-2

**Published:** 2024-05-15

**Authors:** Marco Waldmann, Marc Bohner, Long-Quan R. V. Le, Anna Baghnavi, Bianca Riedel, Michael Seidenstuecker

**Affiliations:** 1 Department of Orthopedics and Trauma Surgery, G.E.R.N. Tissue Replacement, Regeneration & Neogenesis, Medical Center-Albert-Ludwigs-University of Freiburg, Faculty of Medicine, Albert-Ludwigs-University of Freiburg, Hugstetter Straße 55, 79106 Freiburg, Germany; 2 Robert Mathys Foundation RMS, Bischmattstr. 12, 2544 Bettlach, Switzerland

**Keywords:** β-TCP, PRP, Ceramic, Monocyte, Immunofluorescence, Live/Dead, Degradation

## Abstract

β-TCP ceramics are versatile bone substitute materials and show many interactions with cells of the monocyte-macrophage-lineage. The possibility of monocytes entering microporous β-TCP ceramics has however not yet been researched. In this study, we used a model approach to investigate whether monocytes might enter β-TCP, providing a possible explanation for the origin of CD68-positive osteoclast-like giant cells found in earlier works.

We used flow chambers to unidirectionally load BC, PRP, or PPP into slice models of either 2 mm or 6 mm β-TCP. Immunofluorescence for CD68 and live/dead staining was performed after the loading process.

Our results show that monocytes were present in a relevant number of PRP and BC slices representing the inside of our 2 mm slice model and also present on the actual inside of our 6 mm model. For PPP, monocytes were not found beyond the surface in either model.

Our results indicate the possibility of a new and so far neglected constituent in β-TCP degradation, perhaps causing the process of ceramic degradation also starting from inside the ceramics as opposed to the current understanding. We also demonstrated flow chambers as a possible new in vitro model for interactions between blood and β-TCP.

## Introduction

### β-TCP

Beta-tricalcium phosphate (β-TCP) ceramics are widely used, bio-degradable ceramics used in many settings of bone reconstructive surgery. They find application e.g. in anterior cruciate ligament reconstructions [1], interbody fusions [2], as suture anchors [3] or as drug delivery systems [4]. Recent animal studies also indicate an even wider area of use with positive local effects in osteoporotic settings [5, 6] or even cancer treatment [7, 8]. β-TCP can also be used as an ingredient for mixed ceramics such as biphasic calcium phosphates, which are considered the gold standard of bone substitutes in bone reconstructive surgery [9].

Degradability is one of the key features of β-TCP and is believed to start with degradation on the surface of the ceramics, ultimately leading to a replacement with spongy bone [10]. An important factor for bone tissue regeneration is the similarity in chemical composition of β-TCP and native bone [11]. The degradation of β-TCP is primarily a result of chemical dissolution, and consequently, the resorption by osteoclasts plays a subordinate role [12]. It is also worth mentioning that the process of degradation and replacement seems to be less pronounced in humans than it is seen in animal models [13].

### Macrophage-monocyte-lineage

Animal studies demonstrated that the interaction of β-TCP with cells from the monocyte-macrophage lineage leads to increased production of proinflammatory cytokines [7, 14]. Macrophages derived from monocytes also play a pivotal role for the fate of implanted biomaterials leading to either encapsulation or integration [15].

The monocytes themselves are found in peripheral blood and develop within the bone marrow from progenitor cells derived of hematopoietic stem cells [16, 17]. They make up about 10% of leukocytes in peripheral blood and are precursor cells to macrophages and osteoclasts [18].

Some frequently used commercial antibody markers for the monocyte-macrophage lineage are CD68 [19–21], CD14 [21, 22] and CD16 [21]. Expression of CD14 and CD16 can also be used to further classify monocytes into three subsets of either CD14^high^CD16^−^ (classical), CD14^high^CD16^+^ (intermediate), or CD14^low^CD16^+^ (non-classical) monocytes. Each subset has a different half-life time and plays a different role in further cell differentiation. With about 85% of monocytes the CD14^high^CD16^−^ subset is the most prominent and can either differentiate into CD14^high^CD16^+^ monocytes or disappear from circulation after a lifespan of 1-2 days [17, 23].

The CD14^high^ monocytes can differentiate into macrophages, which can be functionally divided into groups of either classically activated M1 or alternatively activated M2 macrophages [24]. M1 macrophages generally play a pro-inflammatory role, whereas M2 macrophages usually have anti-inflammatory effects and lead to tissue regeneration [25]. Depending on the microenvironment, macrophage phenotypes may also alternate between M1 and M2 [26]. In the context of TCP implants, the pro-inflammatory macrophages, together with osteoclasts, could potentially play a role in material resorption via phagocytosis [27], while anti-inflammatory macrophages play an important, but not completely understood role in bone formation and vascularization [28].

Monocytes have also been described to adhere to TCP [29] and lead to the formation of osteoclast-like cells on the surface of ceramics [30]. However, the possibility of monocytes entering microporous β-TCP ceramics has not yet been discussed even after CD68-positive osteoclast-like giant cells with unknown origin were found inside of the ceramics during the degradation process [10]. As a possible explanation for these findings, we hypothesized that peripheral blood monocytes are capable of entering microporous β-TCP ceramics. To prove this idea, we tried to introduce monocytes found in suspensions derived from peripheral blood into the ceramics by applying a mild vacuum via means of a flow chamber.

## Material and methods

### Materials

**Table:** 

**Material**	**Information**
AlexaFluor488 secondary antibody	Sigma-Aldrich, Darmstadt, Germany, SAB4600044, 1:1000
BSA	AppliChem, Darmstadt, Germany, A1391,0025, 1%
Calcein-AM and EthD-III	Biotrend, Cologne, Germany, 30002-T
CD68-primary antibody	Abcam, Cambridge, UK, ab955, 1:100
DAPI	Sigma-Aldrich, Darmstadt, Germany D8417, 1:1000
DMEM/F12	Thermo Fisher, Waltham, USA, 11330057
FCS	Sigma Aldrich, Darmstadt, Germany, S0615, 10%
Filter: Alizarin/Xylenolorange; Cy5&AF647; Tetracyclin; FITC/Cy5 H Dualband Filter	AHF, Tuebingen, Germany
Methanol	Sigma-Aldrich, Darmstadt, Germany, 32213-1L, -20°C
Olympus BX51 fluorescent microscope, equipped with a 10x objective	Olympus, Tokyo, Japan
PBS	Sigma-Aldrich, Darmstadt, Germany,08662
Thermanox™ Coverslip membrane	Thermo Fisher, Waltham, USA, 174985
UV-Lightsource X-Cite Series 120 Q	Excelitas Technologies, Waltham, USA

### β-TCP

The Robert Mathys Foundation (RMS) produced the β-TCP ceramics according to our specifications. A mixture of eighty grams α tricalcium phosphate (α- TCP; Ca3(PO4)2), 20 g tricalcium phosphate (Art. No. 102143, Merck, Switzerland), 60.0 ± 0.2 g solution of 0.2 M Na2HPO4 and 1% polyacrylic acid (Art. No. 81132, Fluka, Switzerland; Mw = 5.1 kDa) was produced. After intensively stirring for 2.5 minutes, the paste was poured into a plastic syringe of 70 mm length and 23 mm diameter and left for 45 minutes. The paste was then covered with 10 mL of phosphate-buffered saline (PBS) (Item No. P5368, Sigma, USA), pH 7.4 solution, and incubated for 3 days at a temperature of 60°C. Afterwards, the green bodies were dried at 60 °C and sintered for 4 h at 1250 °C using a heating and cooling rate of 1 °C/min. The ceramic cylindrical-shaped bodies were now cut to 7 mm diameter and 25 mm length. To remove wear particles and organic residues by combustion, the ceramics were washed in an ethanol bath and calcined at 900 °C [31].

β-TCP ceramics produced with this method were analyzed in other works using environmental scanning electron microscopy (ESEM) and were found to have a mean pore diameter of 4.8 ± 1.2 µm [32] or an average pore radius of 2.1 ± 0.3 µm [33].

β-TCP dowels were sawn into 6 mm or 2 mm thick slices and subsequently placed into an ultrasonic bath starting with 10 minutes in 70% ethanol followed by 10 minutes in distilled water. Afterwards, the slices were autoclaved and ready to be loaded into a flow chamber.

### Sampling

Buffy coats (BC) and EDTA whole blood were provided by the Institute for Transfusion Medicine and Gene Therapy, University Freiburg. BC was used without further preparation and 60 ml EDTA whole blood samples were pooled to further process into either PRP (platelet-rich plasma) or PPP (platelet-poor plasma). Blood counts were performed by the Institute for Clinical Chemistry and Laboratory Medicine, University Freiburg. Analyses were performed on a Sysmex XN-9100 analyzer (Reagent: Cellpack DCL/DS; Sysmex Europe, Norderstedt, Germany). Platelets were measured using the impedance method (resistance measurement). Monocyte counts as part of a differential blood count were determined by flow cytometry as a 6-part differential. BC, PPP, and PRP were analyzed in a 1:10 dilution and pooled EDTA whole blood without dilution.

PRP was created using centrifugation according to Cattaneo et al. 2013 [34]. 60 ml pooled EDTA whole blood was centrifuged at 200 g for 10 minutes (no breaks). The supernatant of the erythrocyte fraction (plasma and the leukocyte+platelet rich middle layer) was then pipetted into a new tube resulting in PRP with an increased monocyte count. The target range for platelet counts was 600-800,000/µl to contrast higher platelet counts in BC.

PPP was meant as a negative control with low monocyte and platelet numbers and created according to Jo et al. 2013 [35]: We centrifuged 60 ml pooled EDTA whole blood at 900 g for 5 minutes (no breaks). Transferred the supernatant of the erythrocyte fraction (plasma and the leukocyte and platelet rich layer) into a new vessel and centrifugated at 1500 g for 15 minutes (no breaks).

Afterwards, the upper 2/3 of the liquid supernatant were pipetted into a new vessel to obtain platelet-poor plasma with a low monocyte count (PPP). To receive even lower platelet counts, the last centrifugation step (1500 g x 15 minutes) was repeated in one of the samples.

### Loading via flow chambers

**Fig. 1:**
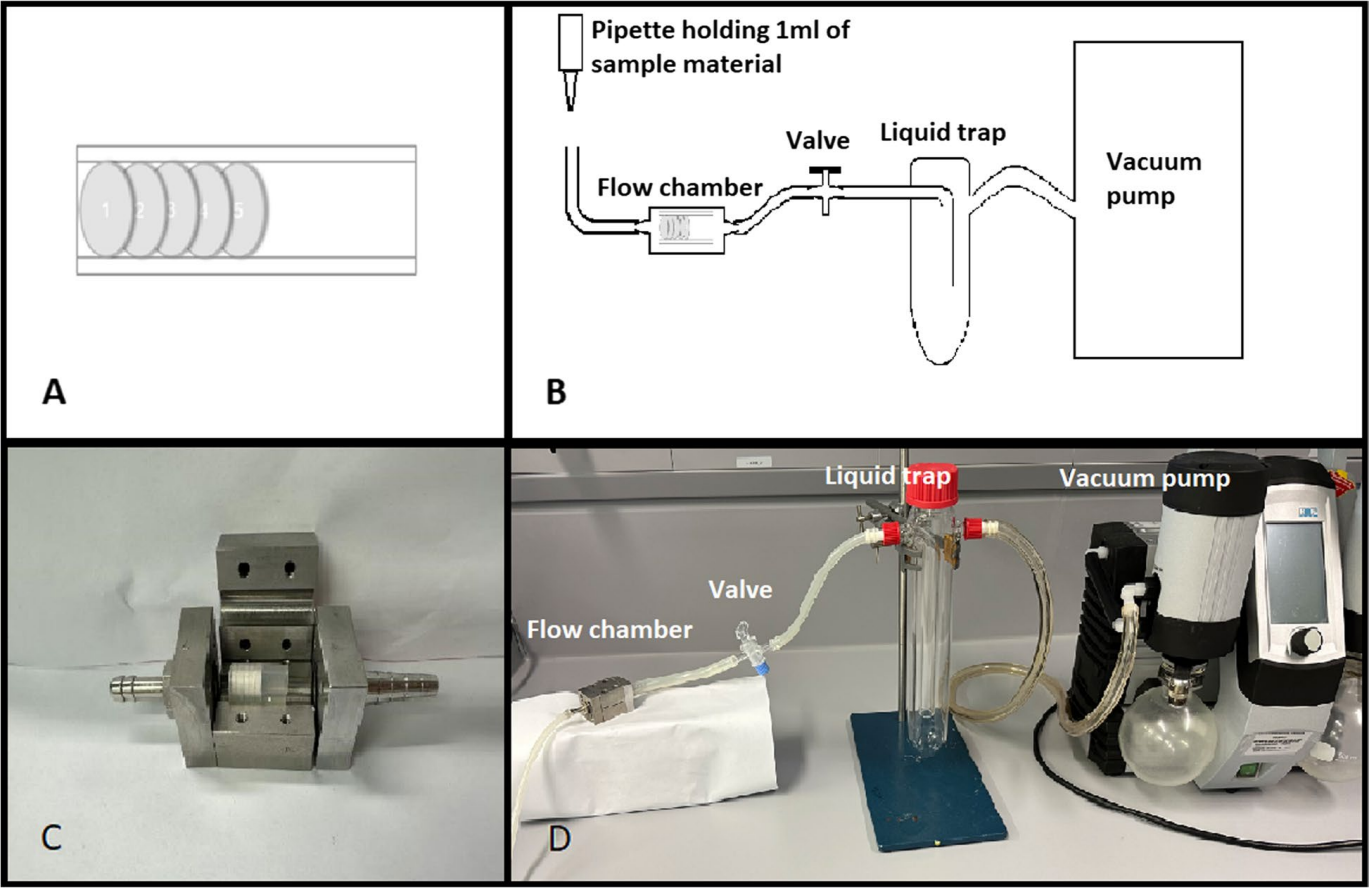
Experimental setup for the loading of flow chambers. Frame **A** shows a schematic of five 2 mm slices loaded into a silicone seal and Frame **C** shows a loaded silicone seal placed into an opened flow chamber. The essential components of the loading process are shown as a schematic representation (Frame **B**) or as an actual build respectively (Frame **D**)

TCP was loaded into stainless-steel flow chambers as described by Seidenstuecker et al. 2015 [36]. After being placed into a silicone seal, five 2 mm slices were put into a flow chamber. The slices are later referred to in accordance with the flow direction of the sample material. Slice 1 is the first slice resembling 0 mm depth, slice 2 with 2 mm depth, slice 3 with 4 mm depth, slice 4 with 6 mm depth and slice 5 as the last slice with 8 mm depth (Fig. 1). Up to four flow chambers were loaded per sample.

Integrity of slices 1 and 5 was visually controlled during the whole loading process to prevent a sample flow through cracks.

After attaching transparent vacuum hoses to both sides of the chamber, a mild vacuum of 350 mbar is applied. When reaching the target pressure, 1 ml sample material of either BC, PRP, or PPP was pipetted into the vacuum hose connecting to the front side of the flow chamber. Normally, loading did not lead to the suspension being completely drawn through the chamber and was terminated approximately 15-20 seconds after the sample came to a standstill. The chamber was now reopened and the silicone seal carrying the 2 mm slices is stored in DMEM/F12 + 10% FCS until further treatment on either the same day (day 0) or the next day (day 1). If a sample tube had to be stored overnight, it was placed inside an incubator at 37 °C.

### CD68-Immunofluorescence

Slices were retrieved from the silicone tube and allowed to dry for 15 minutes. Every slice was separately washed in PBS, fixated in -20 °C Methanol for 10 minutes, blocked in 1% BSA for 60 minutes, and then incubated with CD68-primary antibody over night at 4 °C. On the next day, slices were stained with AlexaFluor488 secondary antibody and counterstained with DAPI at room temperature. Slices were repeatedly washed with PBS between each step.

Monocytes were identified by their size of 15–22 µm [37] combined with CD68-positivity [38] and their origin from peripheral blood as by nature of our samples. Additionally, DAPI-counterstaining had to be in direct relation to the observed CD68 fluorescence for us to assume that the monocytes were intact.

### Live/dead assay

Slices loaded with BC, PRP, or PPP were stained using Calcein-AM and EthD-III at room temperature on either day 0 or day 1. Living cells were identified by green fluorescence and dead cells by red fluorescence. Cells with a size ranging from 18-22 µm were seen as monocytes in the live/dead assay. Cells with a range of 15-17 would technically also qualify as monocytes but would not be distinguishable from eosinophil granulocytes with a size range of up to 17 µm. Cells smaller than 15 µm were valued as granulocytes [37].

A Thermanox™ Coverslip membrane incubated with BC was used as a positive control.

### Microscopy

We performed fluorescence microscopy to examine the front side of each slice immediately after either live/dead or CD68-immunofluorescence staining using an Olympus BX51 fluorescent microscope equipped with a 10x objective. A UV-Lightsource X-Cite Series 120 Q was used for excitation and pictures were taken using filters Alizarin/Xylenolorange and Tetracyclin for live/dead pictures and filters Cy5&AF647 and FITC/Cy5 H Dualband Filter for CD68-immunofluorescence pictures.

### 6 mm slice

To verify that used samples go through the microporous structure of the ceramics and positive results in the 2 mm slice model are no product of samples flowing around the edges of each slice, we also performed loading, immunofluorescence staining and live/dead staining of 6 mm slices for BC, PRP, and PPP. Chambers were loaded with a singular 6 mm slice inside a silicone tube. The slice was cracked open vertically by using pliers before staining. We then analyzed the breaking edge resembling the inside of the slice using fluorescence microscopy in accordance to the 2 mm slices.

## Results

### CD68-Immunofluorescence

**Fig. 2:**
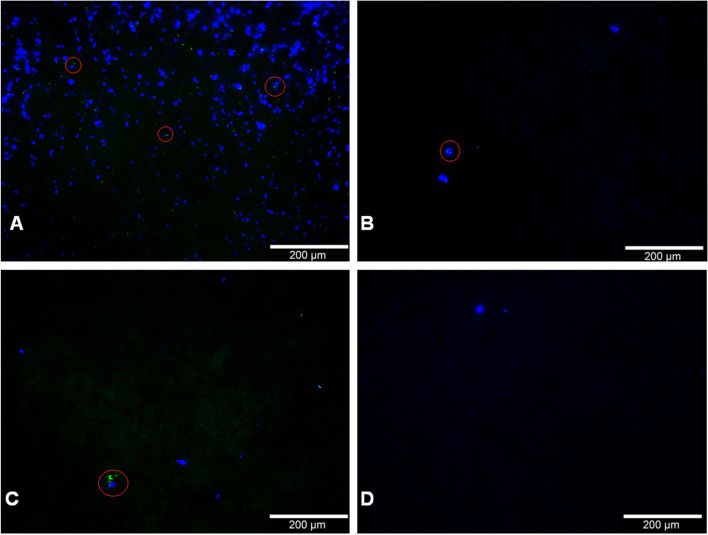
Staining for CD68 (green) with DAPI counterstaining (blue) exemplary for the frontside of a 2 mm slice at 0 mm depth (slice 1) loaded with PRP (**A**), the frontside of a 2 mm slice at 4 mm depth (slice 3) loaded with PRP (**B**), the frontside of a 2 mm slice at 8 mm (slice 5) depth loaded with PRP (**C**), and a the frontside of a 2 mm slice at 0 mm depth (slice 1) loaded with PPP (**C**). Pictures were taken with an Olympus BX51 fluorescent microscope at 10x magnification. Monocytes were highlighted exemplarily with red circles. Filters Cy5&AF647 and FITC/Cy5 H Dualband Filter were used

Immunofluorescence signals resembling CD68-positive cells (Fig. 2) were found on 4 out of 5 analyzed depths for BC and on 3 out of 5 analyzed depths for PRP. Slice 4 (6 mm depth) did not provide any positive cell signals and slice 2 (2 mm depth) was only positive for BC.

The results separated for each slice had positive results on 5/7 for slice 1 (0 mm depth) 3/3 for slice 2 (2 mm depth), 3/7 for slice 3 (4 mm depth), 0/3 on slice 4 (6 mm depth), and 1 out of 7 for slice 5 (8 mm depth) when loaded with BC.

PRP on the other hand had slices with positive results on 6/8 for slice 1 (0 mm depth), 0/2 for slice 2 (2 mm depth), 5/8 for slice 3 (4 mm depth), 0/2 for slice 4 (6 mm depth), and 1/8 for slice 5 (8 mm depth).

The combined outcome for BC and PRP showed 11/15 on slice 1 (0 mm depth), 3/5 on slice 2 (2 mm depth), 8/15 on slice 3 (4 mm depth), 0/5 on slice 4 (6 mm depth), and 2/15 on slice 5 (8 mm depth) with positive results.

For PPP, only slice 1 (0 mm depth) gave positive results while no cells were found on any other slice. With 3/7 positive results, slice 1 (0 mm depth) for PPP reported fewer positive attempts than PRP (6/8) or BC (5/7) (Table 1).

**Table 1:**
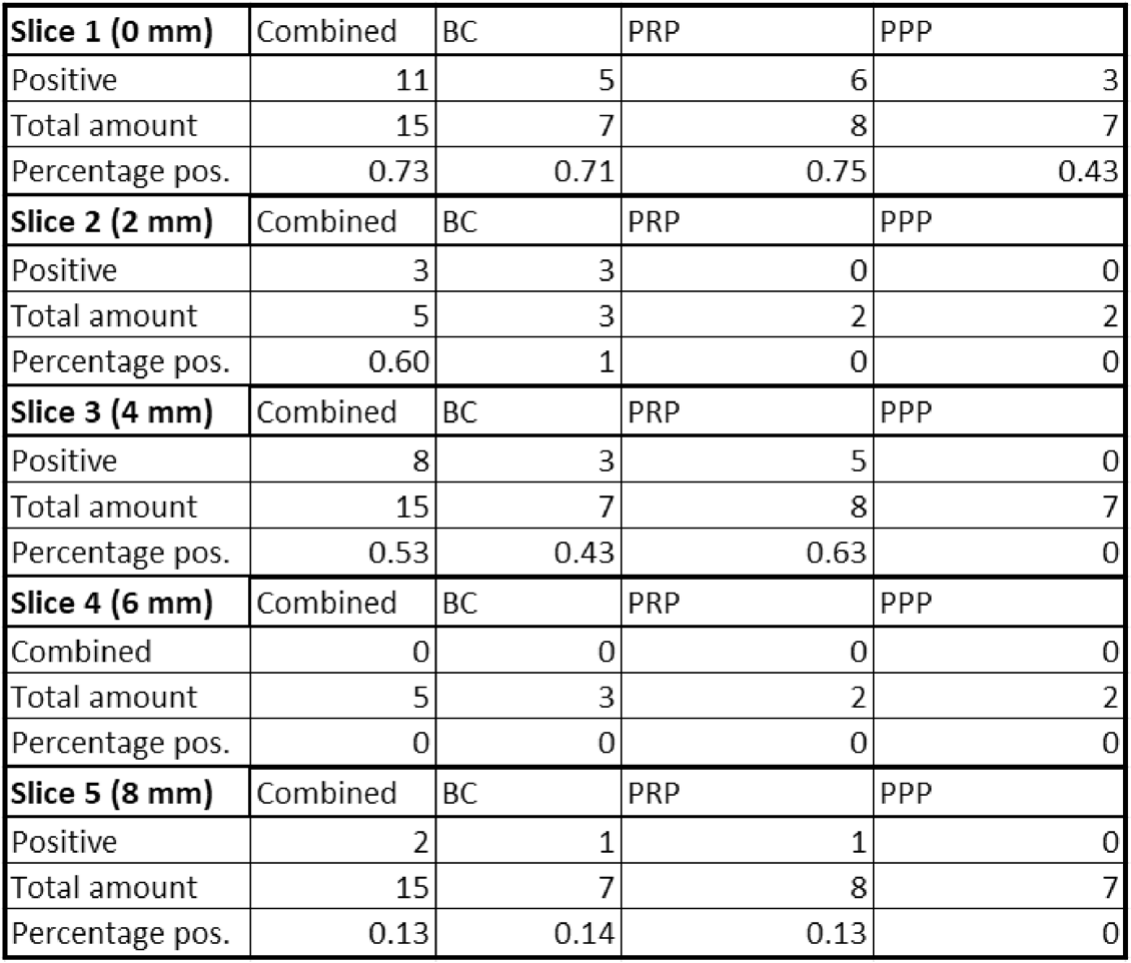
Number of analyzed slices we were able to find at least one positive signal of a CD68-positive cell separated by sample material as well as buffy coat and platelet-rich plasma combined. Each progressing slice number resembles 2 mm increasing depth with slice 1 starting at 0 mm depth. The results are presented in absolute and relative numbers

When split up depending on the day of immunofluorescence staining (Table 2), we found that CD68- positive cells were only detected on depths exceeding 0 mm in samples that were incubated for one day. At least one CD68-positive cell was found on 2/3 PRP slice 3 (4 mm depth) and 1/3 slice 5 (8 mm depth).

**Table 2:**
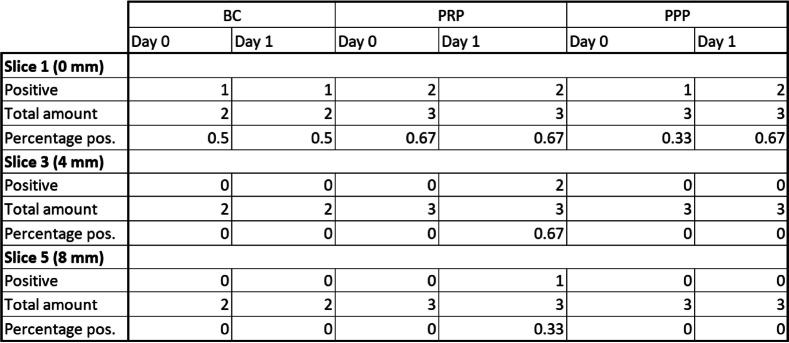
Number of slice 1 (0 mm depth), slice 3 (4 mm depth) and slice 5 (8 mm depth) with at least one positive signal of a CD68-positive cell split up between slices that were immunofluorescence stained starting on the same day as the loading of the vital suspension into the flow chamber happened (Day 0) and slices that were stained after a day of incubation at 37 °C (Day 1). The results are presented in absolute and relative numbers

BC and PPP had no positive results inside the ceramic for slice 3 (4 mm depth) and slice 5 (8 mm depth) on day 0 or day 1.

The results separated for each used vital suspension (Table 3) show positive results on slice 3 (4 mm depth) for at least one slice for every used BC and PRP sample. On slice 5 (8 mm depth) the BC with the highest count of 4794 monocytes/µl has 1/4 positive slices as well as the PRP with the lowest count of 1602 monocytes/µl. The BC with 2004 monocytes/µl and the PRP with 1695 monocytes/µl, respectively, have no positive slices on slice 5 (8 mm depth).

**Table 3:**
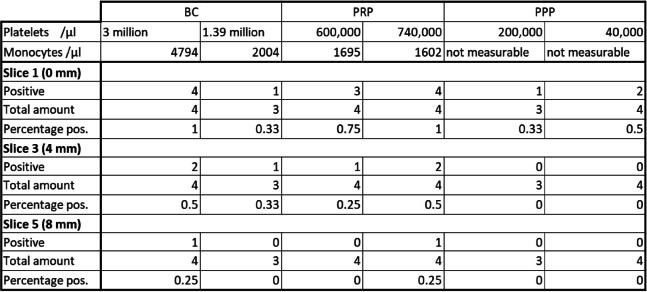
Number of slice 1 (0 mm depth), slice 3 (4 mm depth), and slice 5 (8 mm depth) with at least one positive signal of a CD68-positive cell split up for each vital suspension used in the loading process. The respective platelet and monocyte count for every vital suspension is reported at the top. The results are presented in absolute and relative numbers

In both PPP samples no slices with CD68-positive cells were found inside the ceramic at any inspected depth. However, 1/3 slices for the suspension with 200,000 platelets/µl and 2/4 slices for the suspension with 40,000 platelets/µl on slice 1 (0 mm depth) were positive for at least one cell, even though monocytes were not measurable in blood counts for both samples.

### Live/dead assay

**Fig. 3:**
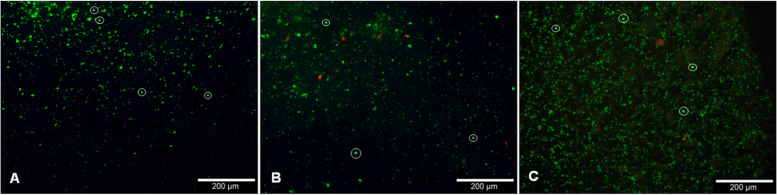
Live/dead staining using Calcein-AM and EthD-III for the breaking edge of a 6 mm slice loaded with BC (**A**), the front side of a 2 mm slice at 4 mm depth loaded with BC (**B**), and a Thermanox™ Coverslip membrane incubated with BC as a control (**C**). Living cells were identified by green fluorescence and dead cells were identified by red fluorescence. Pictures were taken with an Olympus BX51 fluorescent microscope at 10x magnification. Monocytes were highlighted exemplarily with white circles. Filters for Alizarin/Xylenolorange and Tetracyclin were used

Living cells as identified by green fluorescence after live/dead staining made up the vast majority of the cells found on the BC slices that were analyzed on the same day as they were loaded inside the flow chambers while only singular cells were found dead. The majority of the cells had a diameter below <15 µm but also cells with a size of over 18 µm were recognized (Fig. 3).

After slice 3, the cell number drops noticeably but keeps the same size distributions up to slice 5. The number of dead cells seems to increase with further depth/slices (Data not shown). Slices analyzed on the day after being loaded seemed to have slightly more dead cells with up to multiple dead cells per field of view but still showed mainly living cell signals (Fig. 3).

### mm slice

Live/dead staining was positive in 2 out of 2 BC samples. Smaller (<15 µm diameter) live/dead positive cells were observed all the way through the ceramic, with bigger cells (>18 µm) rapidly declining after approximately 2-3 mm inside the ceramic (Fig. 5).

**Fig. 4:**
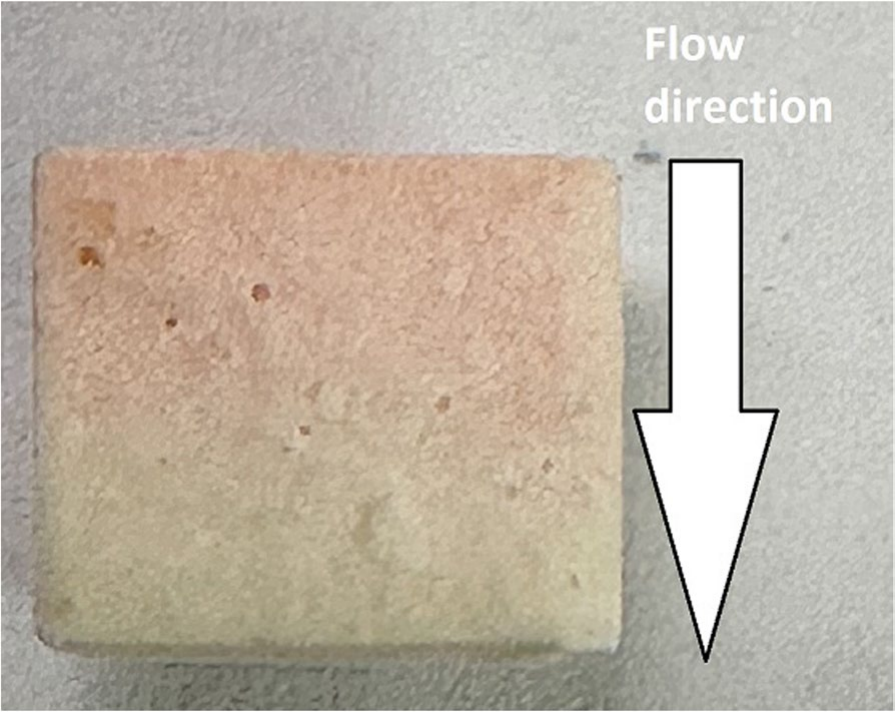
Macroscopic picture of the breaking edge of a 6 mm dowel slice incubated with PRP. The flow direction of PRP during the loading process is implicated by the arrow within the picture

The breaking edges in 2 out of 4 PRP samples and in 0 out of 2 PPP controls showed CD68-positive cells. The stained cells in the positive group entered the ceramic up to a depth of approximately 2.5 mm with no CD68-positive cells reaching halfway or further through the ceramic (Data not shown). The breaking edges of the 6 mm PRP and BC slices were visibly stained red, decreasing in intensity with the flow direction and could be perceived without the help of a microscope (Fig. 4).

## Discussion

The main goal of our experiment was to clarify the origin of CD68-positive (giant-)cells inside of microporous β-TCP ceramics that had been reported in earlier works [10]. As osteoclast-like cells could either origin from the surrounding tissue or peripheral blood monocytes [18], the origin of those cells had yet to be researched and found a possible explanation with the monocytes observed in this study.

The possibility of a RANKL-independent formation of osteoclasts triggered by IL-6 and TNF-α [39] could mean that the monocytes found in this work lead to osteoclast formation even without further interaction with osteoblasts. With monocytes recognized inside the microporous structure, the formation of osteoclasts in similar locations becomes a very real possibility that could impact the understanding of β-TCP degradation, which so far is believed to begin almost exclusively on the surface of the ceramics and is being led by chemical dissolution [30].

**Fig. 5:**
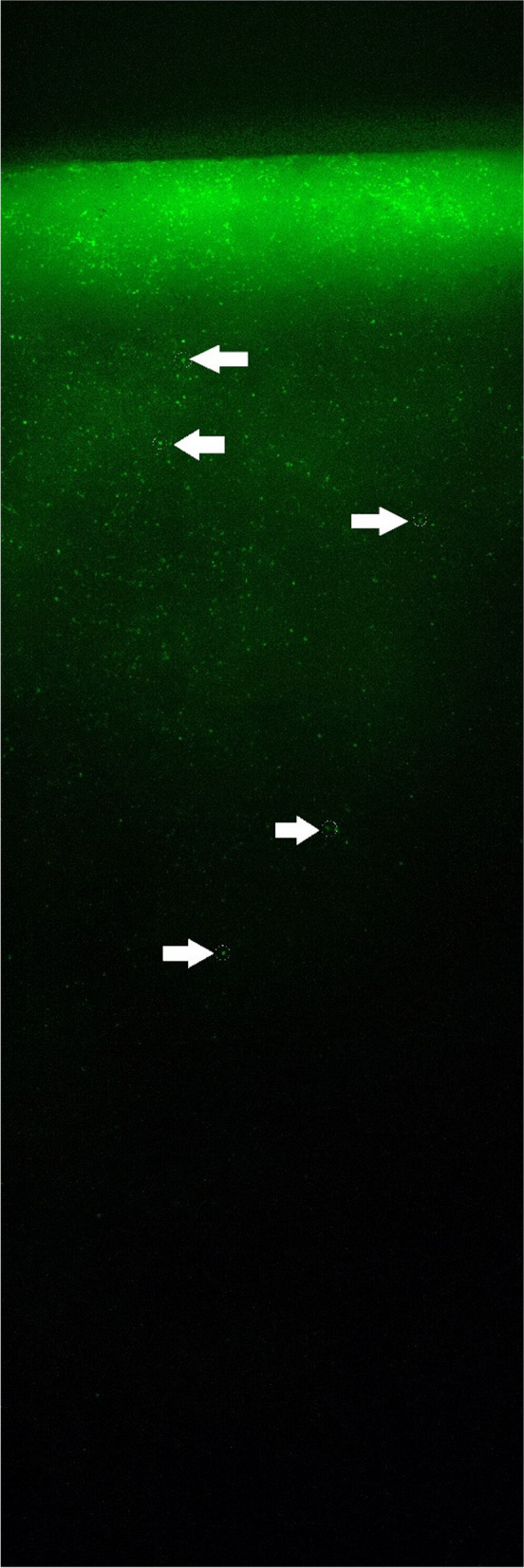
Live/dead staining using Calcein-AM and EthD-III for the vertical breaking edge of a 6 mm slice loaded with BC. Multiple pictures at 10x magnification were merged to show an elongated area starting with the surface which faced towards the flow direction during the loading process shown at the top and continuing according to the flow direction downwards in the picture. The orientation of this Figure matches the arrow shown in Fig. 4. Only the Calcein-AM staining (Green) for living cells is displayed. EthD-III staining for dead cells (red) is not shown in the picture. Pictures were taken with an Olympus BX51 fluorescent microscope at 10x magnification. Monocytes were highlighted exemplarily with white circles and arrows

With BC and PRP respectively showing CD68-positive signals inside our model at various depths (Table 1), our data suggests that the introduction of monocytes from blood-derived vital suspensions inside of β-TCP ceramics is successfully possible. The gradual decrease of positive slices over slice 1, 3 to 5 (Table 1) is also in line with this observation and an expected effect as cells were introduced from just one direction and will reach the furthest β-TCP slice in lower numbers. After slice 3 (4 mm depth), the number of positive slices dropped rapidly (Table 1). A similar phenomenon was observed in our 6 mm slice with a drop off at about 2-3 mm (Fig. 5). The adsorption of proteins to the ceramics surface [40] might increase the resistance so much that further progression into the ceramic was barely possible. The cutting into five slices for our 2 mm slice model probably lowered the resistance shown against the stained cells compared to the 6 mm counterpart and explains the later drop off in cell numbers for the 2 mm model.

The cell structure observed during immunofluorescence sometimes appeared different from the expected phenotype. We assume that this is due to an effect previously described for cells on hydroxyapatite ceramics by Bouler et al. 2017 [9] where only the extremity of the cells and lamellipodia were visible. Another explanation that comes to mind would be deformation experienced by the cells during the vacuum process. Should the vacuum process however have damaged the cells, we would expect high numbers of dead cells in the live/dead assay. But with cell viability being high for monocytes and granulocytes in the live/dead assay (Fig. 3), we deem this possibility as unlikely.

Since monocytes were almost exclusively found inside of the ceramic onwards from day 1, chemotaxis of monocytes triggered by neutrophils [41] may play a bigger role in our experiment than the used vacuum. On the other hand, the amount monocytes seemed to have no recognizable effect on positive outcomes, as the BC sample with the highest positive count had the same outcome as the PRP sample with the lowest count (Table 3). With no difference depending on sample monocyte count, we also did not expect to find any larger deviation between BC and PRP slices and given our results, we see this assumption confirmed. Though for practical reasons, the possibility to use PRP in perioperative settings [42] could prove advantageous should further experiments be considered.

Surprisingly, the PPP samples that were meant as a negative control with no detectable monocyte count in the differential blood count showed positive results on slice 1 (0 mm depth) (Table 3). As our samples however were analyzed in a 1:10 dilution and with an amount of 1 ml PPP used per experiment, a residual number of monocytes is likely to be found even without detection in the differential blood count and explains these findings. But as seen for BC and PRP, the amount of cells progressing into the ceramic gradually decreases (Table 1) and the amount of monocytes found on the PPP slices was not enough to be found on any slices beyond slice 1.

As this work, to our knowledge, is the first one to use the flow chambers described in Seidenstuecker et al. 2015 [36] to examine interactions of β-TCP with blood components, we would also like to propose the idea of loading these flow chambers with various blood suspensions as a potential new model for in-vitro interactions of blood components and β-TCP suitable for monocytes (Fig. 2, Table 1), granulocytes (Fig. 3), and erythrocytes (Fig. 4).

### Limitations

While false-positive results were a real and very possible concern for us, the addition of the 6 mm dowel which was split open only after the experiment gave us certainty that our results in fact resemble cells that went through the ceramic’s pores and were not just products of a faulty experimental setup. Risk of false-positive results was also a reason to lay little focus on slice 2 (2 mm depth) of our experiments as cracks in the first slice could never be completely excluded and would have easily produced false-positives.

With some BC and PRP slices for slice 1 (0 mm depth) giving no positive signal even though high monocyte numbers were observed in the differential blood count (Table. 3), we must also assume that the sensitivity of our immunofluorescence protocol was limited and should be further optimized.

All blood products used in this work are subject to interindividual differences. To reduce the impact of said differences, PPP and PRP samples were created by pooling samples of multiple individuals. BC samples were not available in quantities high enough to allow pooling.

In addition, BC samples used in this work were stored for about 24 hours at room temperature by the Institute for Transfusion Medicine and Gene Therapy, University Freiburg before being further processed, creating a possible distortion of our results.

The loading process via vacuum does not resemble a physiological method and might lead to differences in penetration time and overall cell composition found within the ceramics.

## Conclusion

By demonstrating the viability and character of our cells, we were able to demonstrate the successful integration of human peripheral blood monocytes into the micropores of microporous β-TCP in an in vitro setting, and a yet to be explored role in the formation of osteoclast-like giant cells and thus additional degradation from within the ceramics, as opposed to the current understanding of degradation from the surface of the ceramics alone. This work might therefore help to close the knowledge gap on the origin of these osteoclast-like giant cells and could help for a better understanding of β-TCP degradation behavior, possibly allowing more precise predictions about the degradation in vivo.

Further, we have shown a possible new in vitro model for the interaction of human blood and β-TCP ceramics using flow chambers.

## Data Availability

The datasets used and/or analysed during the current study are available from the corresponding author on reasonable request.
